# Anti-mesothelin vaccine CRS-207 with or without low-dose cyclophosphamide plus chemotherapy as front-line treatment for malignant pleural mesothelioma (MPM)

**DOI:** 10.1186/2051-1426-3-S2-P161

**Published:** 2015-11-04

**Authors:** Raffit Hassan, Evan Alley, Hedy Kindler, Scott Antonia, Thierry Jahan, Anish Thomas, Somayeh Honarmand, Aimee L Murphy, John J Grous, Dirk G Brockstedt

**Affiliations:** 1 Center for Cancer Research at the National Institutes of Health, Bethesda, MD, USA; 2 University of Pennsylvania, Philadelphia, PA, USA; 3 The University of Chicago Medicine, Chicago, IL, USA; 4 H. Lee Moffitt Cancer Center and Research Institute, Tampa, FL, USA; 5 University of California San Francisco, San Francisco, CA, USA; 6 Aduro Biotech Inc., Berkeley, CA, USA; 7 Aduro Biotech Inc., Hopedale, MA, USA

## Background

CRS-207 is live-attenuated, double-deleted *Listeria monocytogenes* (LADD) engineered to express the tumor-associated antigen mesothelin which is highly expressed in malignant pleural mesothelioma (MPM). CRS-207 stimulates potent innate and adaptive immunity and in combination with chemotherapy may act synergistically to alter the tumor environment to be more susceptible to immune-mediated killing. Preliminary data of 32 patients who received CRS-207 in combination with pemetrexed/cisplatin showed 60% partial responses and 94% disease control[1]. Low-dose cyclophosphamide (Cy) in combination with LADD improved immune and anti-tumor responses and overall survival in preclinical studies.

## Methods

Up to 60 subjects are planned to be enrolled in 2 mutually exclusive, sequential cohorts at 5 clinical trial sites. Patients must be chemotherapy-naïve, have unresectable MPM, good performance status (ECOG 0 or 1) and adequate organ function. Eligible patients in Cohort 1 receive 2 prime vaccinations with CRS-207 (1 × 10^9^ CFU; 250 mL IV over 2 hours) 2 weeks apart, followed by up to 6 cycles of pemetrexed (500 mg/m^2^) and cisplatin (75 mg/m^2^) 3 weeks apart and 2 CRS-207 boost vaccinations 3 weeks apart. Subjects are followed every 8 weeks until disease progression. Clinically stable patients continue CRS-207 maintenance vaccinations every 8 weeks. Patients in Cohort 2 receive low-dose Cy (200 mg/m^2^) 1 day prior to each CRS-207 vaccination. Objectives of the study are safety, immunogenicity, objective tumor responses and tumor marker kinetics.
